# X Chromosome Inactivation during Grasshopper Spermatogenesis

**DOI:** 10.3390/genes12121844

**Published:** 2021-11-23

**Authors:** Alberto Viera, María Teresa Parra, Sara Arévalo, Carlos García de la Vega, Juan Luis Santos, Jesús Page

**Affiliations:** 1 Laboratorio A-113, Departamento de Biología, Facultad de Ciencias, Universidad Autónoma de Madrid, 28049 Madrid, Spain; mayte.parra@uam.es (M.T.P.); sara.arevalom@estudiante.uam.es (S.A.); carlos.delavega@uam.es (C.G.d.l.V.); 2 Departamento de Genética, Facultad de Biología, Universidad Complutense de Madrid, 28040 Madrid, Spain; jlsc53@bio.ucm.es; 3 Laboratorio C-001, Departamento de Biología, Facultad de Ciencias, Universidad Autónoma de Madrid, 28049 Madrid, Spain

**Keywords:** sex chromosomes, meiosis, transcriptional activity, grasshopper, *Eyprepocnemis plorans*, MSCI

## Abstract

Regulation of transcriptional activity during meiosis depends on the interrelated processes of recombination and synapsis. In eutherian mammal spermatocytes, transcription levels change during prophase-I, being low at the onset of meiosis but highly increased from pachytene up to the end of diplotene. However, X and Y chromosomes, which usually present unsynapsed regions throughout prophase-I in male meiosis, undergo a specific pattern of transcriptional inactivation. The interdependence of synapsis and transcription has mainly been studied in mammals, basically in mouse, but our knowledge in other unrelated phylogenetically species is more limited. To gain new insights on this issue, here we analyzed the relationship between synapsis and transcription in spermatocytes of the grasshopper *Eyprepocnemis plorans*. Autosomal chromosomes of this species achieve complete synapsis; however, the single X sex chromosome remains always unsynapsed and behaves as a univalent. We studied transcription in meiosis by immunolabeling with RNA polymerase II phosphorylated at serine 2 and found that whereas autosomes are active from leptotene up to diakinesis, the X chromosome is inactive throughout meiosis. This inactivation is accompanied by the accumulation of, at least, two repressive epigenetic modifications: H3 methylated at lysine 9 and H2AX phosphorylated at serine 139. Furthermore, we identified that X chromosome inactivation occurs in premeiotic spermatogonia. Overall, our results indicate: (i) transcription regulation in *E. plorans* spermatogenesis differs from the canonical pattern found in mammals and (ii) X chromosome inactivation is likely preceded by a process of heterochromatinization before the initiation of meiosis.

## 1. Introduction

Meiosis is a specialized cell division that secures the maintenance of chromosome number across generations in sexually reproducing organisms. Meiosis is characterized by a single round of DNA replication followed by two successive chromosome segregation events, leading to the formation of haploid gametes. The faithful segregation of chromosomes must be preceded by three steps in the behavior of homologous chromosomes: (i) recognition and pairing (ii) synapsis, mediated by the assembly of the synaptonemal complex, and (iii) recombination, leading to the reciprocal exchange of chromosomal regions. Synapsis and meiotic recombination are interrelated processes occurring during prophase-I, although their chronological schedule has been found to differ among model organisms [1]. As a rule, recombination initiates at the beginning of prophase-I by the generation of programmed DNA double strand breaks (DSBs) by the enzyme SPO11 [2,3,4,5,6]. DSBs are followed by the rapid phosphorylation of some histone variants such as H2AX, H2Av [7,8], or H2B [9], in the regions surrounding a given DSB. These epigenetic modifications have been related with the recruitment of repair factors for facilitating the repair efficiency of DSBs [7,10]. The meiotic repair of DSBs is mainly performed through the homologous recombination pathway that involves DNA strand invasion and exchange between homologous chromosomes [11]. In most eukaryotes, these events are mediated by the recombinases RAD51 and DMC1 [12,13,14]. Their action is crucial for homology search and synapsis completion in pachytene and also for the proper formation of crossovers, whose cytological manifestation are chiasmata [15,16,17,18,19,20,21,22,23].

Progression of synapsis and recombination during prophase-I are accompanied by changes in the organization of chromatin [24,25], epigenetic marks [26,27,28,29] and transcription activity [30,31,32,33,34]. In eutherian mammals, the rate of transcription found in spermatogonia decreases as meiosis initiates, but it is reactivated during pachytene. Subsequently, transcriptional activity is again repressed at metaphase-I and recovered in differentiating spermatids. This pattern of transcriptional activity indicates that during leptotene and zygotene, when synapsis is progressing, transcription remains in low levels and that a noticeable burst of transcription occurs in pachytene when synapsis has been achieved completely [33]. However, the chromosomal regions that fail to complete synapsis remain in a silenced state and undergo a process called meiotic silencing of unsynapsed chromatin (MSUC) [35,36,37,38], which prevents the reactivation of unrepaired/unsynapsed chromosomes. During mammalian male meiosis, this process is especially conspicuous for sex chromosomes, which present large unsynapsed regions in most species and manifests as a specific inactivation process called meiotic sex chromosome inactivation (MSCI) [39,40,41]. Thus, MSUC/MSCI are intimately related to the progression of DNA recombination/repair and the achievement of pairing and synapsis of homologous chromosomes during prophase-I.

Studies in species from other different taxa, particularly insects, have revealed deviations from the well-characterized patterns of the regulation of transcription and MSUC/MSCI displayed by eutherian mammals. For instance, transcriptional activity in grasshoppers, moths, true bugs and scorpions is rather abundant in early stages of meiosis [42,43,44,45,46,47,48,49,50,51]. Interestingly, many of these species present a sex chromosomal determinism of the type XX for females and X0 for males, which is especially interesting. The fact that the single sex chromosome in males behaves as a univalent during meiosis can shed light about the way in which the regulation of transcription and the extent of MSUC/MSCI during meiosis occur in these organisms.

In previous reports, we have revealed the interdependence between the patterns of recombination and pairing/synapsis of homologous chromosomes in spermatocytes of several grasshopper species [23,52,53,54,55]. In the present study, we analyzed the progression of chromosome pairing/synapsis and recombination in relation to transcriptional activation/inactivation in spermatocytes of the grasshopper *E. plorans*. Thus, we could compare the behavior of autosomal homologous chromosomes, which achieve full synapsis, with that of the single X chromosome that is an asynaptic and achiasmatic univalent. Our results demonstrate that, from leptotene up to diakinesis, transcriptional activity is maintained in most autosomal regions, while the X chromosome invariably is inactive. Moreover, we determined that the X chromosome most likely enters meiosis in a pre-inactivated state that is not reversed during prophase-I.

## 2. Materials and Methods

Adult males of *E. plorans* (Orthoptera: Acrididae) were collected in San Juan de Alicante (Spain) from natural populations, under the appropriated licenses and conditions (authorization 3126/nm from Conselleria de Agricultura, Medio Ambiente, Cambio Climático y Desarrolllo Rural; Generalitat Valenciana). The chromosome complement of this species is composed of 24 chromosomes in females (2n = 22 + XX) and 23 chromosomes in males (2n = 22 + X). A testicular biopsy from each specimen was fixed in freshly prepared 3:1 ethanol-glacial acetic acid and squashed in a drop of 50% glacial acetic acid. The chromosomal number was scored by the analysis of at least 10 diplotene/metaphase I cells under phase contrast microscopy in order to detect the presence of B chromosomes. For this study, we selected the individuals without B chromosomes. Afterward, testes were removed and processed as described below. Twenty-five individuals were employed in this study.

### 2.1. Squashing and Spreading of Seminiferous Tubules

After removing the testes, seminiferous tubules were cleaned in PBS (137 mM NaCl, 2.7 mM KCl, 10.1 mM Na_2_HPO_4_, 1.7 mM KH_2_PO_4_, pH 7.4) and processed for either squashing or spreading. Most observations were performed onto squashed spermatocytes since this methodology allows for the precise 3D location of different structures within a cell. For squashing, we followed the technique previously described [56,57]. Briefly, seminiferous tubules were fixed in freshly prepared 2% formaldehyde in PBS containing 0.1% Triton X-100 (Sigma, Marlborough, MA, USA). After 5 min, several seminiferous tubule fragments were placed with a drop of fixative on a slide previously coated with 1 mg/mL poly-L-lysine (Sigma). Tubules were then minced with tweezers and subsequently squashed. Then, slides were frozen in liquid nitrogen and the coverslip removed. We also employed spreading of spermatocytes since the cells are projected into a single focal plane, facilitating their visualization. Spreading was performed by a drying-down technique based on that previously described [58], with slight modifications [55]. Briefly, the proximal region of tubules was dissected with an entomological pin and removed to avoid the massive presence of spermatids and spermatozoa. Tubules were then homogenized in 100 mM sucrose in distilled water and macerated at room temperature for 15 min. Spermatocytes were simultaneously spread onto slides and fixed with 1% paraformaldehyde in distilled water containing 0.15% Triton X-100. Preparations were dried for 2 h in a moist chamber, washed with 0.08% Photo-Flo (Kodak, Rochester, New York, USA) in distilled water and air-dried.

### 2.2. Immunofluorescence Microscopy

After performing the spread or squash procedures, preparations were rinsed three times for 5 min in PBS and incubated overnight at 4 °C with the corresponding primary antibodies diluted in PBS, all of them previously tested in insect spermatocytes. To detect the cohesin subunit SMC3, we employed either a polyclonal rabbit anti-SMC3 antibody (Chemicon International, Temecula, CA, USA, AB3914) raised against a synthetic peptide from human SMC3 [23], or the K987 rabbit polyclonal antisera (kindly provided by Dr. Barbero) [53], raised against a synthetic peptide corresponding to the carboxy-terminal amino-acid sequence of human SMC3, both at a 1:30 dilution. A rabbit polyclonal anti-RAD51 antibody (Oncogene Research Products, La Jolla, CA, USA, Ab-1, PC130), generated against recombinant HsRad51 protein, was used at a 1:50 dilution [23]. To detect γ-H2AX, we used a monoclonal mouse antibody (Upstate, Burlington, MA, USA, 05-636) at a 1:1000 dilution [23]. Histone H3 trimethylated at lysine 9 (H3K9me3) was revealed with a rabbit polyclonal serum (Abcam, Cambridge, UK, ab-8898) at a 1:100 dilution [55]. RNA polymerase II phosphorylated at serine 2 (p-RNApol-II) was revealed with a mouse monoclonal antibody (Abcam, Cambridge, UK, 24758) at a 1:100 dilution [51]. Following three washes in PBS, the slides were incubated for 30 min at room temperature with the corresponding secondary antibodies. The secondary antibodies used were donkey anti-rabbit IgG (Jackson ImmunoResearch Laboratories, West Grove, PE, USA) at a 1:100 dilution and goat anti-mouse IgG (Jackson ImmunoResearch Laboratories, West Grove, PE, USA) at a 1:100 dilution, both of them were conjugated with Texas Red or fluorescein isothiocyanate (FITC). Finally, a donkey anti-mouse IgM conjugated with DyLight 594 (Jackson ImmunoResearch Laboratories, West Grove, PE, USA) was used to reveal the p-RNApol-II antibody. The slides were subsequently rinsed in PBS, and counterstained for 3 min with 5 µg/mL DAPI (4′, 6-diamidino-2-phenylindole). After a final rinse in PBS, the slides were mounted in VECTASHIELD (Vector Laboratories, Burlingame, CA, USA) and sealed with nail polish. For the double-immunolabeling experiments in which the two primary antibodies were generated in the same host species (SMC3 with either RAD51 or H3K9me3), we proceeded as previously described [59,60]. In these cases, slides were first incubated with the anti-SMC3 antibody for 1 h at room temperature, rinsed three times for 5 min in PBS, and incubated overnight at 4 °C with a DyLight 488-conjugated goat Fab’ fragment anti-rabbit IgG (Jackson ImmunoResearch Laboratories, West Grove, PE, USA) at a 1:100 dilution in PBS. Afterward, slides were rinsed six times for 5 min in PBS, incubated with either anti-RAD51 or anti-H3K9me3 for 1 h, rinsed three times for 5 min in PBS, and then incubated with a donkey anti-rabbit IgG (Jackson ImmunoResearch Laboratories, West Grove, PE, USA) at a 1:150 dilution and goat anti-mouse IgG (Jackson ImmunoResearch Laboratories, West Grove, PE, USA) at 1:150 dilution.

### 2.3. Feulgen–Rossenbeck Reaction

Testes were extracted and fixed in freshly prepared 3:1 ethanol:glacial acetic acid and stored at −20 °C until further usage. Complete testes were processed following the conventional Feulgen–Rossenbeck reaction procedure [61]. After the cytochemical procedure, seminiferous tubules were squashed in a drop of 50% glacial acetic acid, and after freezing the slides in liquid nitrogen, the coverslip was removed. Slides were air-dried and mounted in Eukitt (Sigma, Marlborough, MA, USA).

### 2.4. Histological Sections

For histological sections, testes were fixed by immersion in Bouin’s solution for 24 h. After standard washes and dehydration, Paraplast-embedded tissue blocks were cut in 3 μm thick sections with a Reichert microtome. Finally, sections were stained with conventional Mallory’s trichrome stain.

### 2.5. Image Acquisition and Processing

Observations were performed using an Olympus BX61 microscope (Olympus, Hamburg, Germany) equipped with a motorized Z-axis and epifluorescence optics. Single images or image stacks across complete cells/nuclei were captured with an Olympus DP71 digital camera controlled by CellF Imaging System (Münster, Germany) under fixed capture conditions in order to facilitate the comparison among the intensity of the signals. Images were analyzed, pseudocolored and processed using the public domain ImageJ software (National Institutes of Health, Bethesda, MD, USA; http://rsb.info.nih.gov/ij, accessed on 15 June 2020). Ultimate figures were processed with Adobe Photoshop 7.0 software.

### 2.6. Fluorescence Quantification

Quantification of p-RNApol-II fluorescence was calculated by the integrated density of fluorescence of complete individual nuclei using ImageJ. All images were captured under identical conditions and the projections of complete nuclei were obtained. DAPI staining of each nucleus was employed to generate a binary mask and to calculate the nuclear area. Then, the integrated density of fluorescence for p-RNApol-II was scored. Additionally, in the same image, three background fluorescence measurements were performed. Fluorescence intensity was calculated in each nucleus as the corrected total nuclear fluorescence. Corrected total nuclear fluorescence = integrated density of fluorescence—(area of nucleus X mean fluorescence of background readings). For each cell type, 10 nuclei were analyzed. For statistical analyses, we performed an ANOVA test (confidence level: 95%) and Tukey’s multiple comparisons test. Data were presented by a scatter plot using GraphPad Prism 6.0 software.

## 3. Results

### 3.1. Transcriptional Activity in E. plorans Spermatocytes and Spermatids

**Figure 1:**
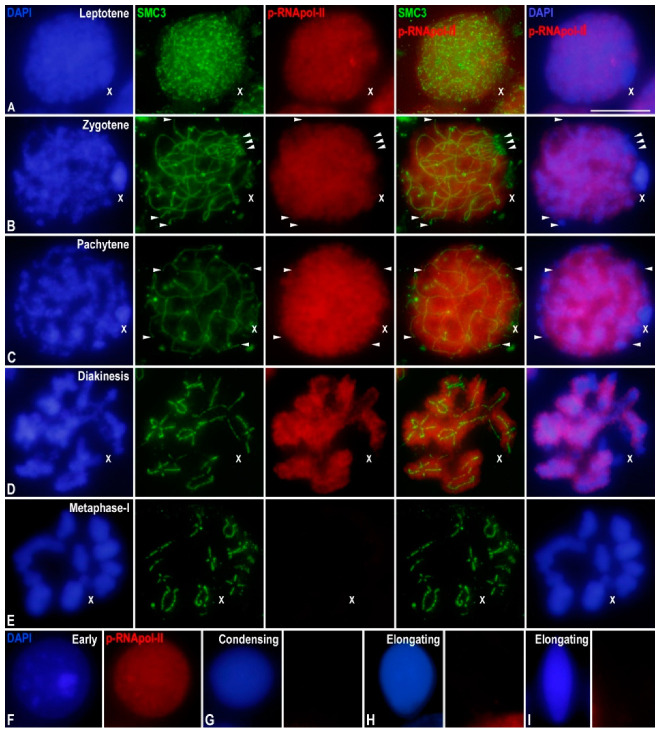
p-RNApol-II distribution in *E. plorans* spermatocytes and spermatids. Projections of stack images acquired across *E. plorans* squashed spermatocytes (**A**–**E**) and spermatids (**F**–**I**), in the stages indicated, stained with DAPI (blue) and double immunolabeled for SMC3 (green) and p-RNApol-II (red). p-RNApol-II labelling slightly increased as meiosis progressed, it being extremely reduced at the sex chromosome and at autosomal ends. The position of the sex (X) chromosome is indicated. White arrowheads in (**B**,**C**) depict autosomal chromosome ends. Scale bar: 10 μm.

In order to determine the transcriptional activity in *E. plorans* spermatocytes, we performed double immunolabeling of SMC3 as a marker of cohesin axes that allows for the assessment of synapsis progression, and hence, spermatocyte staging [23], and RNA polymerase II phosphorylated at serine 2 (p-RNApol-II) as an indicator of transcription. At leptotene, thin SMC3 cohesin axes occupied the entire nucleus, while p-RNApol-II faintly labeled the nucleus (Figure 1A). During zygotene, SMC3 cohesin axes started to pair and produced thicker filaments representing synapsed autosomal segments (Figure 1B and Figure S1A). In these nuclei, the intensity of p-RNApol-II labeling increased on both synapsed and unsynapsed autosomal regions (Figure 1B). Interestingly, p-RNApol-II was absent, or almost absent, from a heteropycnotic body as revealed by DAPI staining. This body was frequently found at the periphery of the nucleus and corresponded to the X chromosome (Figure 1B). On the other hand, the end of the SMC3 axes of some autosomes presented a reduction in the p-RNApol-II labeling (Figure 1B). At pachytene, synapsis was completed and autosomal bivalents presented single and thick SMC3 cohesin axes (Figure 1C and Figure S1A). In contrast, the X chromosome univalent showed a thinner SMC3 axis (Figure 1C and Figure S1A). p-RNApol-II labeling covered the whole nucleus, except the region occupied by the X chromosome (Figure 1C). Moreover, the labeling seemed reduced at the autosomal ends (Figure 1C). During diplotene and diakinesis, characterized by the condensation of the chromatin and the zigzag morphology of SMC3 labeling between the sister chromatids of chromosomes (Figure 1D), p-RNApol-II labeling was present in the autosomal bivalents, but absent from the sex chromosome (Figure 1D). At metaphase-I, SMC3 labeling persisted at the interchromatid domain of chromosomes, while p-RNApol-II labeling became not detectable (Figure 1E). This result was also obtained in later stages of both the first and second meiotic divisions (not shown). Finally, early spermatids showed p-RNApol-II labeling (Figure 1F), which was absent in both condensing (Figure 1G) and elongating spermatids (Figure 1H,I).

In summary, p-RNApol-II in *E. plorans* spermatocytes was detected during leptotene, increased from zygotene up to diakinesis, and ceased from metaphase-I onward. Remarkably, the X chromosome and some autosomal regions were invariably inactive throughout meiosis.

### 3.2. Epigenetic Marks of Transcriptional Inactivation in E. plorans Spermatocytes

**Figure 2:**
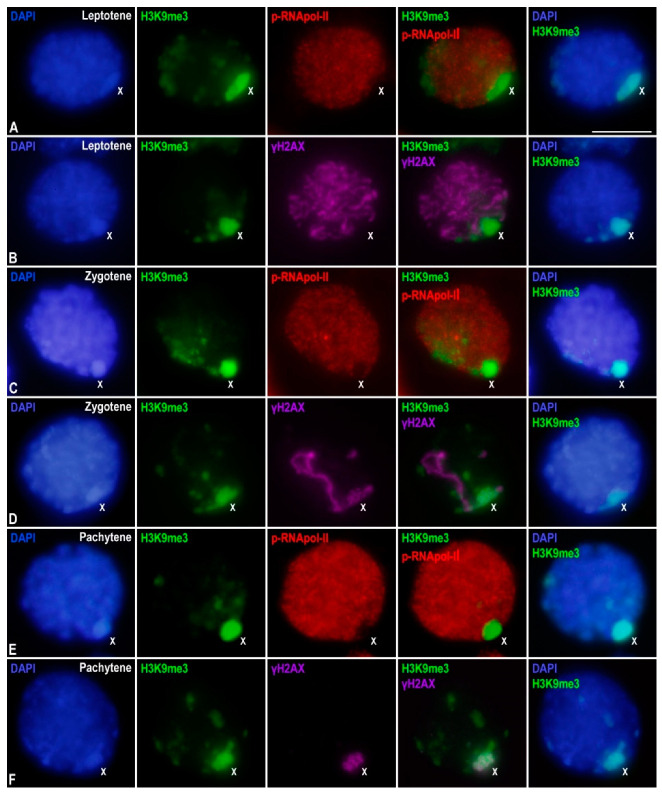
X chromosome inactivation in *E. plorans* prophase-I spermatocytes. Projections of stack images acquired across *E. plorans* squashed spermatocytes, in the stages indicated, stained with DAPI (blue) and double immunolabeled for H3K9me3 (green) and p-RNApol-II (red in (**A**,**C**,**E**)) or γH2AX (purple in (**B**,**D**,**F**)). The position of the sex (X) chromosome is indicated. White arrowheads in (**B**,**C**) depict autosomal chromosome ends. Scale bar: 10 μm.

In order to identify some of the factors involved in the inactivation of the X chromosome during prophase-I in *E. plorans*, we performed immunolabeling of p-RNApol-II and two epigenetic chromatin modifications related to MSUC/MSCI, namely histone H3 trimethylated at lysine 9 (H3K9me3) and histone H2AX phosphorylated at serine 139 (γ-H2AX). In leptotene, H3K9me3 covered the whole sex chromosome and some discrete accumulations were also found over some autosomal regions (Figure 2A,B). As above- mentioned, p-RNApol-II labeling was absent from the X chromosome (Figure 2A). On the other hand, γ-H2AX labeled the nucleus extensively, indicating massive DNA DSBs generated at the onset of meiosis (Figure 2B). Interestingly, at this stage, γ-H2AX did not label the X chromosome (Figure 2B). As previously reported [53], this is encompassed by the absence of RAD51 in the X chromosome of this species (Figure S1A).

During zygotene, H3K9me3 remained accumulated at the X chromosome and other discrete areas of autosomes (Figure 2C,D), which in turn, were devoid of p-RNApol-II labeling (Figure 2C). γ-H2AX did not cover the whole nucleus. In fact, it was only detectable at the unsynapsed autosomal regions and at the X chromosome (Figure 2D). Although both H3K9me3 and γ-H2AX were accumulated over the X chromosome, they presented strikingly different distributions: H3K9me3 was extensively distributed over the chromatin, while γ-H2AX appeared as a ribbon overlaying the position of the cohesin axis (Figure 2D) [23,53]. During pachytene, H3K9me3 persisted over the chromatin of the X chromosome and some discrete autosomal regions scattered in the nucleus (Figure 2E,F), which showed reduced p-RNApol-II signal (Figure 2E). γ-H2AX was restricted to a ribbon in the X chromosome (Figure 2F). Therefore, our results indicate that during prophase-I, the inactive state of the X chromosome and discrete autosomal regions are accompanied, at least, by the accumulation of H3K9me3 and γ-H2AX.

Furthermore, to better identify the autosomal regions that showed intense H3K9me3 labeling and were devoid of p-RNApol-II, we performed the immunolabeling of SMC3 and H3K9me3 on spread spermatocytes, as this technique projects cells into a single focal plane. We found that these regions were positioned at both ends of the SMC3 axis and corresponded to chromosome ends (Figure S1B). A similar labeling was previously reported in other insect species [51,55,62], most probably representing telomeric/subtelomeric heterochromatin at chromosome ends, or the pericentromeric heterochromatin at the centromeric end.

Finally, we analyzed p-RNApol-II and H3K9me3 distribution in spermatids. We observed the labeling of p-RNApol-II through the nucleus and that of H3K9me3 at certain discrete regions, which, in turn, were intensively stained with DAPI (Figure S1E). In later spermatids (Figure S1F,G) the decrease in p-RNApol-II signals was accompanied by a huge increase in H3K9me3 labeling. Unfortunately, and as a consequence of the high compaction of chromatin, the X chromosome or other chromosomal domains could not be distinguished in spermatids.

### 3.3. The X Chromosome Inactivation Occurs in Premeiotic Cells

**Figure 3:**
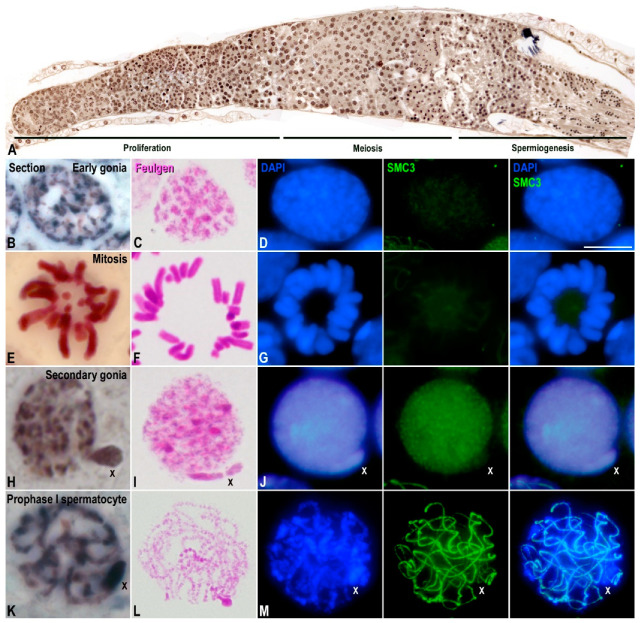
Characterization of *E. plorans* spermatogonia. Projections of stack images acquired across *E. plorans* histological sections (**A**,**B**,**E**,**H**,**K**), squashed spermatogonia (**C**,**D**,**F**,**G**,**I**,**J**), and spermatocytes (**L**,**M**) stained with Feulgen-stain (**C**,**E**,**I**,**L**) or stained with DAPI (blue) and immunolabeled for SMC3 (green) (**D**,**G**,**J**,**M**). The position of the sex (X) chromosome is indicated. Scale bar: 5 μm.

Since the X chromosome seemed to be inactive throughout meiosis in *E. plorans*, we wondered whether this inactivated state and the accompanying epigenetic marks would already be present in premeiotic spermatogonial cells. For this purpose, we characterized the populations of spermatogonia found in *E. plorans*. Grasshopper testes are arranged by multiple compact seminiferous tubules (follicles) that are finally connected to the ejaculatory duct. Each seminiferous tubule is polarized, presenting a closed distal end where proliferation of spermatogonia occurs, a middle zone where spermatocytes undergo meiosis and a proximal zone where spermiogenesis takes place (Figure 3A). Cells maintain cytoplasmic bridges and are arranged into cysts that synchronously progress throughout spermatogenesis. Consequently, cysts move from the distal to the proximal zone of a tubule during their progression along the different stages of spermatogenesis, allowing their identification. In histological sections, we identified two populations of interphase spermatogonia by means of their relative position in the tubule and their morphological characteristics. The first population was located at the distal region of tubules and presented round/oval nuclei with chromatin knobs in which the X chromosome did not show any differential feature (Figure 3B). Mitotic stages were found interspersed among cysts presenting early interphase spermatogonia (Figure 3E). These cell populations were followed by cysts containing secondary spermatogonia showing round nuclei with a conspicuous chromatin protuberance that corresponded to the X chromosome (Figure 3H) [63].

Due to these chromatin features, we could discern the two spermatogonia populations and spermatocytes in squashed preparations after either Feulgen–Rossenbeck (Figure 3C,F,I,L) or DAPI staining (Figure 3D,G,J,M). Moreover, spermatogonia were also differentiated by SMC3 labeling, since in early stages, they did not show significant signals (Figure 3D,G), while in secondary spermatogonia, SMC3 labeling was uniformly scattered over the nucleus (Figure 3J). Likewise, primary spermatocytes were distinguished by the labeling of cohesin axes with SMC3 (Figure 3M).

**Figure 4:**
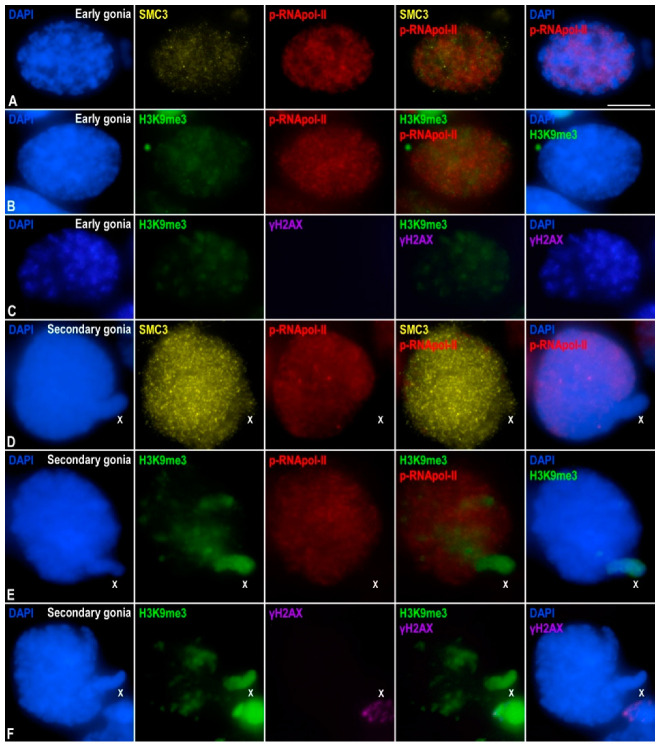
Transcriptional activity in *E. plorans* spermatogonia. Projections of stack images acquired across *E. plorans* squashed spermatogonia, in the stages indicated, stained with DAPI (blue) and double immunolabeled for SMC3 (yellow) and p-RNApol-II (red) (**A**,**D**); H3K9me3 (green) and p-RNApol-II (red) (**B**,**E**); or H3K9me3 (green) and γH2AX (purple) (**C**,**F**). The position of the sex (X) chromosome is indicated. Scale bar: 5 μm.

The next step was the analysis of the distribution of p-RNApol-II, H3K9me3, and γ-H2AX in premeiotic cells. Early spermatogonia showed faint p-RNApol-II marks distributed through the entire nucleus (Figure 4A) and faint H3K9me3 labeling without evident accumulations at any region (Figure 4B), and no γ-H2AX marks (Figure 4C). Consequently, the X chromosome did not seem to be specifically inactivated in early spermatogonia, at least by the same epigenetic histone modifications that operate during meiosis. Secondary spermatogonia also presented p-RNApol-II distributed in the whole nucleus, but the signal was extremely reduced or absent from the X chromosome (Figure 4D). In contrast, huge accumulation of H3K9me3 over the sex chromatin (Figure 4E) and some autosomal regions, mostly corresponding to positively heteropycnotic chromatin after DAPI staining, were observed (Figure 4E). Unfortunately, the entanglement of SMC3 cohesin axes in these cells (Figure 4D) impeded us in undoubtedly determining whether H3K9me3 accumulations at autosomes corresponded to telomeres, as observed in spermatocytes. No γ-H2AX signal was found in secondary spermatogonia (Figure 4F), indicating that, at this stage, X chromosome heterochromatinization and inactivation did not depend on γ-H2AX. Overall, these results indicated that the X chromosome appeared inactive in secondary spermatogonia, and this was accompanied by the deposition of epigenetic histone modifications, some of which were also present in spermatocytes.

### 3.4. Transcription Is Maintained in the Transition from Premeiotic Cells to Spermatocytes

**Figure 5:**
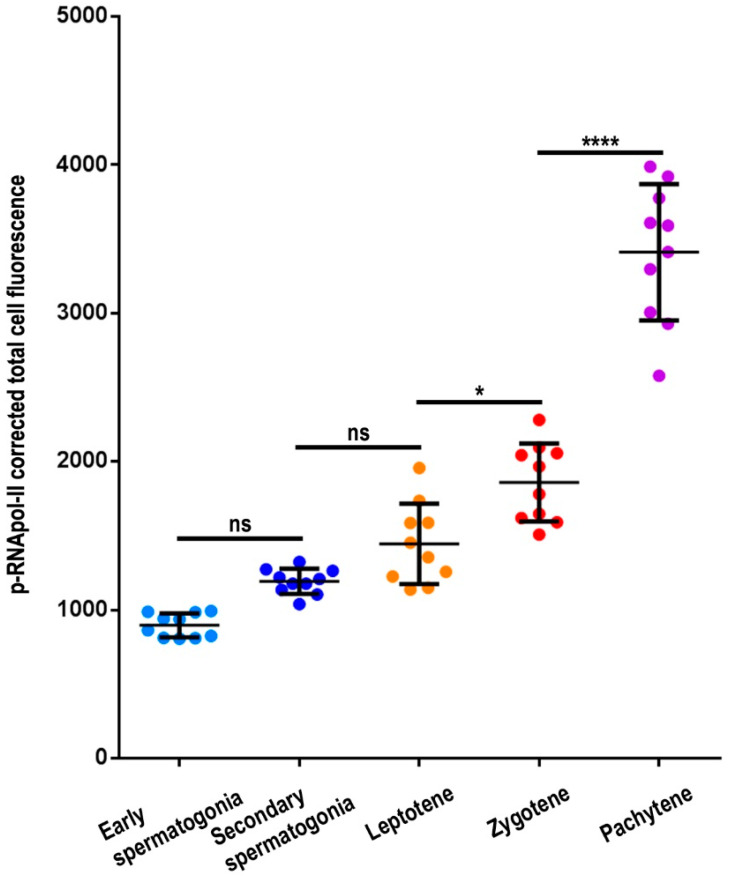
p-RNApol-II quantification in *E. plorans* spermatogonia and spermatocytes. Scatter plot analyses of corrected total nuclear fluorescence of p-RNApol-II in a given spermatogonia or spermatocyte at the indicated stages (n = 10 for each stage). The median value and standard deviations are depicted. Statistical significance was assessed using an ANOVA test (*p* < 0.0001) and Tukey’s multiple comparisons test (ns, not significant; *, *p* < 0.1; ****, *p* < 0.00001).

Taking into account that the overall intensity of p-RNApol-II seemed to increase from early spermatogonia up to prophase-I spermatocytes (Figure S1D and Video S1), we performed fluorescence quantification of the nuclear p-RNApol-II labeling. Our analysis demonstrated a progressive and significant (3.8-fold) increase in p-RNApol-II intensity from early spermatogonia up to pachytene (ANOVA; *p* < 0.0001) (Figure 5). Tukey’s multiple comparison test showed no statistical differences between early and secondary spermatogonia or between secondary spermatogonia and leptotene. However, the differences were significant between leptotene and zygotene (*p* < 0.01) and much more evident (1.8-fold) between zygotene and pachytene (*p* < 0.0001) (Figure 5). Altogether, these data revealed that the amount of p-RNApol-II labeling found in early spermatocytes was comparable to that of spermatogonial premeiotic cells. These findings indicate that there was no inactivation at the beginning of meiosis, and that there was a subsequent burst in transcription from the zygotene onward.

## 4. Discussion

The regulation of transcription activity during meiosis has attracted increasing attention in the past years [31,64,65]. The fact that both coding and noncoding (including miRNas) sequences are actively expressed during most of the first meiotic prophase implies a striking difference to the pattern characterizing mitosis. Preliminary studies on the regulation of transcription activity during meiosis were performed in the 1960s [32,43,46]. This topic received renewed attention when it was realized that in spermatocytes of eutherian mammals, transcriptional activity was tightly coupled to both DNA repair and chromosome synapsis, which are characteristic of meiotic prophase-I [35,38,66]. Overall, transcription drops at the beginning of meiosis and then burst during pachytene [32,33,34], and the regions that fail to complete DNA repair or recombination remain inactive and trigger an MSUC/MSCI response [35,36,37,38,67]. Thus, the disruption of either recombination or synapsis hampers the whole normal program of transcription and the epigenetic transitions that encompass meiosis progression [26]. However, this model might not be completely applied to other model organisms such as *Caenorhabditis elegans* [42] or *Drosophila melanogaster* [47], and neither to several species of grasshoppers [43,46,48] and true bugs [49,51], in which transcriptional activity in early spermatocytes is abundant prior to synapsis achievement.

### 4.1. The Pattern of Meiotic Transcription in Spermatocytes of E. plorans Differs from That Displayed by Eutherian Mammals

Our results indicated that in *E. plorans*, spermatocyte transcription is also active at autosomes in leptotene, long before synapsis completion, as proposed in other grasshopper species [43,46]. Considering that the rate of transcription in leptotene is comparable to that found in spermatogonial premeiotic cells, a specific inactivation of transcription at the onset of meiosis was not plausible. These results were consistent with a previous report in *E. plorans* showing that histone H3 acetylated in lysine 9 (H3K9ac), an epigenetic mark related to high levels of transcription, was broadly present at autosomes throughout prophase-I stages [68]. Consequently, DNA repair, asynapsis and transcriptional activity in this species were not mutually excluding processes, as proposed in mammals [33,69]. Our data and previous reports in insects and other invertebrate species (see above) indicated that transcription regulation in these groups during prophase-I differed from that of mammals. Although it could be difficult to decipher the intrinsic mechanisms generating these differences, an epigenetic basis might be envisioned. In mouse, leptotene and zygotene spermatocytes extensively accumulate several epigenetic marks related to gene silencing and heterochromatin formation. Some examples of these marks are the histone modifications H3K9me3, H3K4me and γH2AX [26,29,33,39,70]. In contrast, in *E. plorans*, H3K9me3 was mostly absent from the chromatin of autosomes during the entire prophase-I, and the location of this histone modification was restricted to both the centromeric and distal chromosome ends. This fact could facilitate a permissive state for transcriptional activity. According to this proposal, the enrichment of H3K9me3 at telomeres was also accompanied by a local reduction in p-RNApol-II. It must be noted that the accumulation of H3K9me3 at telomeres, which has also been related to heterochromatin formation [70], has previously been reported in other grasshopper [55] and hemipteran species [51,62]. It would be interesting to determine whether these heterochromatic regions in *E. plorans* corresponded to those previously described to have a reduced accumulation of H3K9ac [68]. Moreover, the presence of H3K9me3 at heterochromatic regions of autosomes in secondary spermatogonia might indicate that the transcriptional inactivation of these regions is established in premeiotic cells and maintained in spermatocytes, as it was suggested in true bugs [51].

On the other hand, it is noticeable that in *E. plorans*, transcription activity in early meiosis is compatible with high γH2AX levels. Aside from its participation in DNA DSBs signaling, the presence of this histone mark has been associated with transcription down-regulation after DNA damage, triggered by the DSB-responsive kinase ATM [39,71,72]. Indeed, γH2AX is supposed to be one of the main markers of MSUC/MSCI [40,41]. However, γH2AX and transcription may not be completely excluding. Although relatively low, transcription in mammalian spermatocytes at leptotene and zygotene is not completely abolished [34,64]. Additionally, it is possible that the DNA damage response in *E. plorans* is not as intense as in mammals. Considering that H2AX phosphorylation only affects the area surrounding the DSBs, it is likely that many genomic regions are not affected by the inactivation reaction. Thus, γH2AX might not be a factor sufficient for triggering whole transcription inactivation during early meiosis. In fact, other epigenetic factors such as H3K9me3 could be more relevant, as suggested for mouse meiosis [33].

In conclusion, it seems that *E. plorans* and other species deviate from the canonical behavior described in mammals with regard to the relationship between DNA damage, synapsis and transcription. As above-mentioned, each particular combination of epigenetic modifications may result in a specific pattern of transcription regulation [26].

### 4.2. X Chromosome Heterochromatinization and Inactivation

MSCI in eutherian mammals is thought to be a general process that refers to the transcriptional silencing of genes on the X and Y sex chromosomes in spermatocytes. This chromosome-wide silencing is mediated by the condensation state exhibited by sex chromosomes. However, MSCI is incomplete in dogs, perhaps as a consequence of extensive, but transient, self-synapsis of the X chromosome, in association with rapid completion of meiotic DSB repair [73]. On these grounds, and from an evolutionary point of view, it would be interesting to know whether this model extends to other biological systems. Monotremes have been key to understanding the evolution of MSCI in mammals because their sex chromosomes have homology to both the chicken Z and the future therian X chromosome. Platypus prophase-I spermatocytes maintain low transcription rates and lack γH2AX, the hallmark of epigenetic MSCI modifications [74]. Likewise, there is no evidence for MSCI in either synapsed or unsynapsed ZW chromosomes of chicken [75]. These results suggest that meiotic silencing of sex chromosomes evolved in mammals after the divergence of monotremes, presumably as a result of the differentiation of the therian X and Y sex chromosomes.

Other model organisms also deviate from the eutherian pattern. MSCI in *C. elegans* is mediated by deposition of histone H3 dimethylated at lysine 9 [76]. Examination of other *Caenorhabditis* species revealed diverse H3 lysine 9 methylation patterns on the X, suggesting that the sex chromosome epigenome evolves rapidly [77]. Moreover, it has been reported that in *C. elegans* unsynapsed X segments are transcribed in early meiocytes from mutant males and hermaphrodites with bisected X chromosomes [78]. On the other hand, in *D. melanogaster* the existence of MSCI is a matter of debate [79]. Recent findings, based on measuring all genes or a set of broadly expressed genes in testis, indicate that the single X is specifically inactivated in primary spermatocytes due to failed activation of RNA polymerase II by phosphorylation of serine 2 and 5 [80]. In contrast, genes on the single Y chromosome become maximally active in primary spermatocytes.

In *E. plorans*, we found that the X chromosome was inactive during the entire prophase-I. This was accompanied by the accumulation of H3K9me3 (this work) and the absence of H3K9ac [68]. This epigenetic pattern was also observed in secondary (but not primary) spermatogonia. These findings suggest that the X chromosome could enter meiosis in an inactive state, and this would rely, at least partially, on the same epigenetic features in both premeiotic and meiotic cells. Since primary spermatogonia did not display a conspicuous X chromosome inactivation, the facultative heterochromatinization of this chromosome is likely to occur only in spermatogonia that are about to enter meiosis [63]. As shown here and previously, grasshopper leptotene spermatocytes did not evidence γH2AX labeling at the X chromosome [23,53], while it was extensively present at autosomes. This mark was only detected at the sex chromosome from zygotene onward. Moreover, the X chromosome was also devoid of RAD51 foci (this work) [23,53]. A similar finding was reported in asynaptic sex chromosomes of true bugs [51]. These results suggested that, in these species, sex chromosomes might be somehow protected against the induction of endogenous DSBs occurring in early meiosis. It is likely that the heterochromatic organization of sex chromosomes in grasshoppers and true bugs, before meiosis initiation, could hamper the access of the DSBs machinery, which requires open chromatin conformation and specific epigenetic modifications [1,11]. A similar mechanism has been proposed for the protection of the Y chromosome in mammalian meiosis [69,81]. Alternatively, phosphorylation of H2AX upon DSBs production could be delayed. In this sense, irradiation studies in mammalian spermatogenesis have reported that heterochromatic regions showed a weaker and delayed response to exogenous DSBs [82]. If this was the case, one would expect that some DNA repair proteins, such as RAD51 loaded onto the sex chromosomes in a later prophase-I stage. However, this did not occur in *E. plorans* nor in other grasshopper and true bug species [23,51,53,54,55]. Although alternative mechanism of DSBs repair, not involving RAD51, have been suggested for achiasmate sex chromosomes in *C. elegans* [83] and some mammals [84], their incidence in insects remains to be demonstrated. Therefore, we think that premeiotic heterochromatinization of the X chromosome likely prevents or reduces DSB production.

Regardless of the causes of the delayed phosphorylation of H2AX in the X chromosome, it is possible that the accumulation of γH2AX from zygotene onward could confer an additional strengthening of MSCI. In eutherian mammals, both unsynapsed autosomes and sex chromosomes subjected to MSUC and MSCI, respectively, accumulate a variety of proteins, histone modifications and non-coding RNAs [35,36,41,67,85,86,87], some of which are incorporated sequentially throughout prophase-I [33,69]. Although many of the proteins involved in MSCI in mammals have not been tested in insects, it is possible that H3K9 methylation and H2AX phosphorylation constitute a core of inactivation signatures that could be shared by a wide range of organisms.

The meiotic inactivation of sex chromosomes has been considered one of the processes that are critical for the success of meiosis in male mammals [40,88,89]. This was attributed to the expression of executory genes carried by sex chromosomes, mainly the Y chromosome, when MSCI is hampered [90,91]. Although the situation described here is not entirely comparable to that of mammals, in which sex chromosomes show variable degrees of homologous synapsis, the premeiotic inactivation and heterochromatinization of the single X chromosome reveal the importance of transcription in the synaptic process and perhaps in the adequate completion of meiosis. Further studies on a variety of species with achiasmate sex chromosomes might determine whether these mechanisms are also represented in other groups.

## Data Availability

Not applicable.

## Supplementary Materials

The following are available online at https://www.mdpi.com/article/10.3390/genes12121844/s1, Figure S1: Additional immunolabellings in *E. plorans* spermatocytes and spermatids, (A) Projections of stack images acquired across *E. plorans* squashed spermatocytes stained with DAPI (blue) and double immunolabeled for SMC3 (green) and RAD51 (red) in leptotene (L), zygotene (Z) and pachytene (P); (B) Pachytene spread spermatocyte double immunolabeled for SMC3 (green) and H3K9me3 (red), white arrowheads depict autosomal chromosome ends. The image on the right column depicts a 200% magnification of a selected bivalent; (C) Projections of stack images acquired across *E. plorans* squashed early (E) and secondary spermatogonia (S) and spermatocytes in leptotene (L), zygotene (Z) and pachytene (P) stained with DAPI (blue) and immunolabeled for SMC3 (green); (D) Projections of stack images acquired across *E. plorans* squashed early (E) and secondary spermatogonia (S) and a pachytene spermatocyte (P) stained with DAPI (blue) and immunolabeled for H3K9me3 (green) and p-RNApol-II (red); (E–G) Projections of stack images acquired across *E. plorans* squashed early (E), condensing (F) and elongating spermatids (G) stained with DAPI (blue) and immunolabeled for H3K9me3 (green) and p-RNApol-II (red). White arrowheads in (E) depict the location of heterochromatin. The position of the sex (X) chromosome is indicated. Scale bars in A–D, 10 μm; scale bar in E, 5 μm. Video S1: Comparison of spermatogonia and a pachytene spermatocyte. 3D reconstruction of stack images acquired across *E. plorans* squashed early (E) and secondary spermatogonia (S) and a pachytene spermatocyte (P) stained with DAPI (blue) and immunolabeled for H3K9me3 (green) and p-RNApol-II (red). This reconstruction corresponds to the same field showed in Figure S1D. The position of the sex (X) chromosome is indicated.
