# Supplementary figures and images for: X Chromosome Inactivation during Grasshopper Spermatogenesis
Source: Genes (Basel). 2021 Nov 23;12(12):1844. doi: 10.3390/genes12121844 (PMC8700825; doi:10.3390/genes12121844)

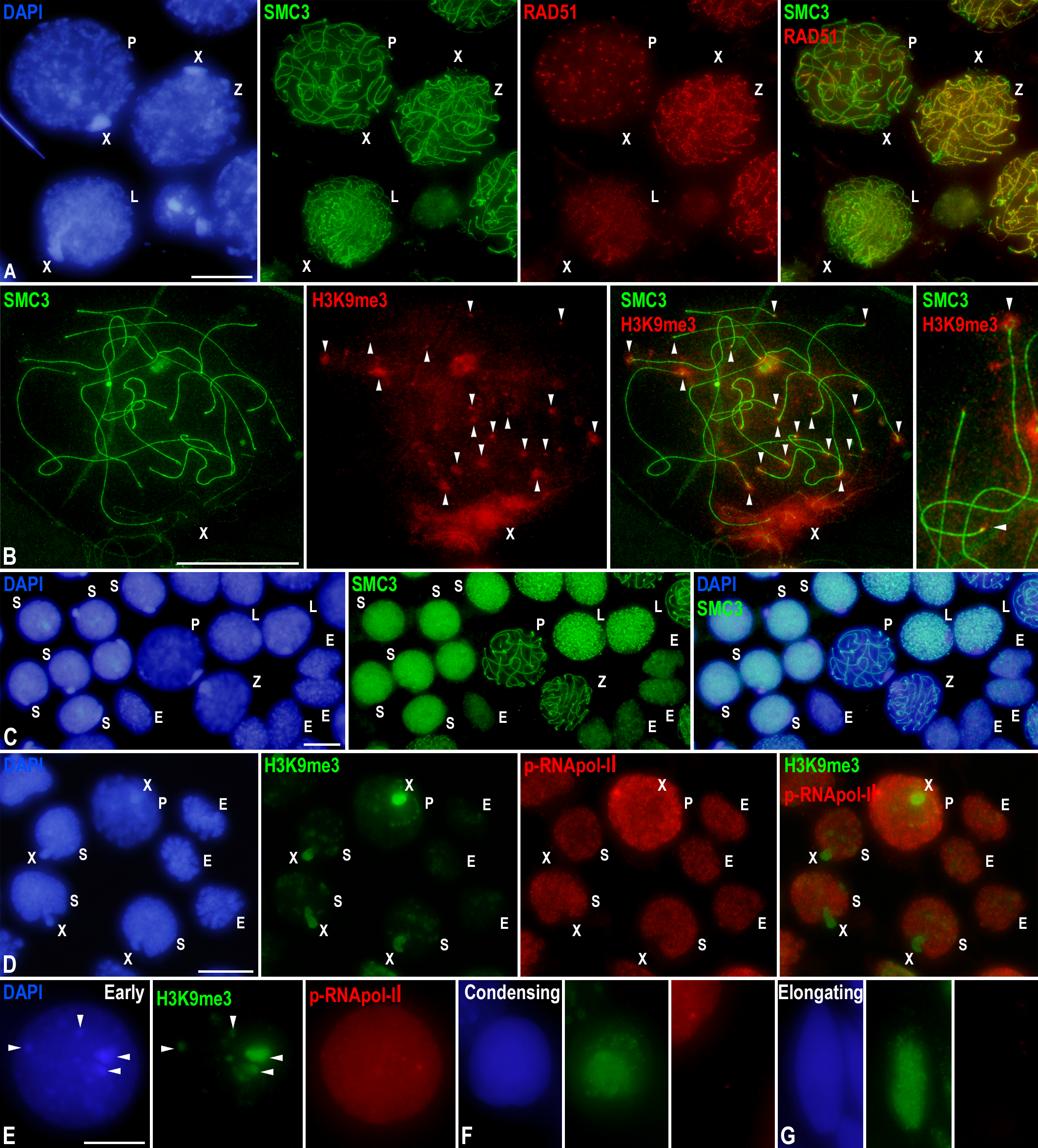

Supplement: Supplementary file 1 [file genes-12-01844-s001.zip › Figure S1.tif]
